# RabGEF1/Rabex-5 Regulates TrkA-Mediated Neurite Outgrowth and NMDA-Induced Signaling Activation in NGF-Differentiated PC12 Cells

**DOI:** 10.1371/journal.pone.0142935

**Published:** 2015-11-20

**Authors:** See-Ying Tam, Jennifer N. Lilla, Ching-Cheng Chen, Janet Kalesnikoff, Mindy Tsai

**Affiliations:** Department of Pathology, Stanford University School of Medicine, Stanford, California, United States of America; Indiana University School of Medicine, UNITED STATES

## Abstract

Nerve growth factor (NGF) binds to its cognate receptor TrkA and induces neuronal differentiation by activating distinct downstream signal transduction events. RabGEF1 (also known as Rabex-5) is a guanine nucleotide exchange factor for Rab5, which regulates early endosome fusion and vesicular trafficking in endocytic pathways. Here, we used the antisense (AS) expression approach to induce an NGF-dependent sustained knockdown of RabGEF1 protein expression in stable PC12 transfectants. We show that RabGEF1 is a negative regulator of NGF-induced neurite outgrowth and modulates other cellular and signaling processes that are activated by the interaction of NGF with TrkA receptors, such as cell cycle progression, cessation of proliferation, and activation of NGF-mediated downstream signaling responses. Moreover, RabGEF1 can bind to Rac1, and the activation of Rac1 upon NGF treatment is significantly enhanced in AS transfectants, suggesting that RabGEF1 is a negative regulator of NGF-induced Rac1 activation in PC12 cells. Furthermore, we show that RabGEF1 can also interact with NMDA receptors by binding to the NR2B subunit and its associated binding partner SynGAP, and negatively regulates activation of nitric oxide synthase activity induced by NMDA receptor stimulation in NGF-differentiated PC12 cells. Our data suggest that RabGEF1 is a negative regulator of TrkA-dependent neuronal differentiation and of NMDA receptor-mediated signaling activation in NGF-differentiated PC12 cells.

## Introduction

Nerve growth factor (NGF) is a member of the family of neurotrophins which also include brain derived growth factor (BDNF) and neurotrophin-3 (NT-3) [1,2]. These neurotrophins are important for the survival, development, and function of neurons in the central and peripheral nervous systems and they exert their effects through their interactions with specific tyrosine kinase receptors: TrkA (NGF), TrkB (BDNF, NT-3), TrkC (NT-3) [3,4]. The molecular mechanisms by which NGF elicits its effects on neuronal differentiation have been intensively studied using the rat adrenal pheochromocytoma cell line, PC12 cells. Upon NGF stimulation, these cells undergo morphological and biochemical changes, resulting in the differentiation to a sympathetic neuron-like phenotype with neurite outgrowth [5]. The stimulation of TrkA receptors expressed on PC12 cells by NGF leads to the endocytosis and trafficking of NGF/TrkA complexes and the formation of signaling endosomes [6]. NGF-mediated signaling is then transmitted retrogradely through axonal transport of signaling endosomes containing NGF, TrkA, and activated signaling intermediate factors such as ERK-kinases [7–9]. These signaling events result in the induction of neurite outgrowth, a hallmark in PC12 differentiation that is characterized by formation of filamentous actin containing spikes followed by growth and extension of long neurite processes [5]. Rab5, a small GTPase known to be involved in the regulation of early endosome fusion and vesicular trafficking in the endocytic pathways [10], is localized in signaling endosomes that contain the endocytosed NGF-TrkA complexes [7,11,12]. It has been proposed that the inhibition of Rab5 activity by TrkA-associated RabGAP5 promotes the diversion of TrkA-containing endocytic vesicles to the formation of signaling endosomes, leading to the propagation of NGF-mediated signaling and neurite outgrowth [12].

Among the downstream signaling cascade intermediates that mediate the NGF-induced cellular responses, several families of small GTPases are critical for the cellular responses to extracellular stimulations and the extensive remodeling of the cytoskeleton. It is well known that the Ras GTPase and its downstream effector ERK-kinase signaling pathways are activated upon the binding of NGF to TrkA, leading to neuronal differentiation [13]. In addition, Rac1 and Cdc42, members of the Rho family of GTPases, have been shown to play a critical role in promoting actin polymerization and cytoskeletal changes in axonal growth, such as neurite outgrowth in NGF-differentiated PC12 cells [14–18]. Recent studies have also implicated Rac1 as an essential signaling factor that mediates retrograde TrkA trafficking and retrograde NGF-induced survival signaling [19,20]. Moreover, the activation of Rac1 by NGF has been shown to be associated with the physical interaction of active Ras with Tiam1, a Rac1-specific guanine nucleotide exchange factor (GEF) [21]. On the other hand, NGF-induced Rac1 recruitment to cell surface sites for cytoskeletal remodeling is associated with concomitant decrease in levels of RhoA activity [22]. RhoA is another member of the Rho family of GTPase and its activation has been shown to inhibit neurite outgrowth and to induce neurite retraction in NGF-differentiated PC12 cells [23].

RabGEF1 (also know as Rabex-5) was initially characterized as a GEF for Rab5 by forming a stable complex with Rabaptin-5, a Rab5 effector, leading to the coupling of Rab5 activation and effector recruitment [24]. We previously identified the mouse ortholog of RabGEF1 in a differential display screen of differentially expressed mRNA transcripts of mouse mast cells activated by aggregation of high affinity IgE receptors (FcεRI) [25]. We showed that RabGEF1-deficient (*Rabgef1* ^-/-^) mice developed spontaneous skin inflammation and that mast cells derived from these animals exhibited delayed receptor internalization, elevated and prolonged intracellular signaling events, and enhanced cytokine and mediator release in response to FcεRI- or c-Kit-dependent activation [25–27]. Amino acid analysis has revealed that RabGEF1 contains several functional domains: (1) A20-like Zn finger (ZnF) domain that exhibits E3 ubiquitin ligase activity and binds to ubiquitin; (2) ‘motif interacting with ubiquitin’ (MIU) domain that binds ubiquitin; (3) Vps9 domain that, together with its adjacent N-terminal helical bundle, comprises the GEF catalytic core; (4) coiled-coil domain with the Rabaptin-5 binding site; (5) C-terminal proline-rich region [26,28–31]. The abilities of RabGEF1 to bind ubiquitin, undergo monoubiquitination, and express E3 ubiquitin ligase activity can contribute to its functions to regulate endosome trafficking via sorting of ubiquitinated cargoes as well as Rab5-dependent endosome fusion. Furthermore, recent studies have shown that RabGEF1 can bind to active Ras to induce Ras ubiquitination and thereby inhibit Ras-dependent ERK activation [32–34].

Considering that RabGEF1 is involved in endosome fusion and vesicular trafficking, several studies have assessed the role of RabGEF1 in neuronal differentiation. Earlier overexpression studies have revealed that Rab5 and RabGEF1 both exert negative regulatory effects on neurite outgrowth induced by NGF in PC12 cells [12]. However, subsequent studies using the shRNA expression approach have shown that RabGEF1 acts as a positive regulator of neurite outgrowth in these NGF-differentiated PC12 cells [35]. Moreover, a recent report has demonstrated that shRNA-mediated knock-down of RabGEF1 or Rab5 inhibits morphogenesis of axons and dendrites in mouse hippocampal neurons [36]. In this study, we used the stable antisense (AS) expression approach to address the role of RabGEF1 in neuronal differentiation using the NGF-differentiated PC12 cell model system. We showed that RabGEF1 negatively regulated neurite outgrowth and other cellular and signaling processes induced by the interaction of NGF with TrkA receptors in these cells. Moreover, RabGEF1 was found to bind to Rac1, and in RabGEF1-AS transfectants expressing reduced levels of RabGEF1, the activation of Rac1 induced by NGF treatment was significantly enhanced, suggesting that RabGEF1 is a negative regulator of NGF-induced Rac1 activation. Furthermore, we showed that RabGEF1 interacted with NMDA receptors by binding to the NR2B subunit and negatively regulated the signaling responses induced by NMDA receptor stimulation in NGF-differentiated PC12 cells. Our data suggest that RabGEF1 is negative regulator of NGF-induced neuronal differentiation and of NMDA receptor-mediated signaling activation in PC12 cells.

## Materials and Methods

### Cell lines and recombinant growth factors

PC12 cells were obtained from ATCC (CRL-1721) and grown in RPMI-1640 supplemented with 10% horse serum (HS) and 5% fetal calf serum (FCS). NGF (2.5S) was purchased from Upstate Biotechnology or Millipore. For NGF-induced differentiation studies, PC12 cells were cultured in RPMI-1640/0.5% FCS with 50 ng/ml or 100 ng/ml NGF.

### Antibodies

Anti—RabGEF1 antibody (mouse anti-Rabex-5 mAb, Clone 27) used in Western blot analyses was obtained from BD Biosciences. Anti-mouse RabGEF1 (Ac-KSER) used in immunoprecipitation studies was prepared by Quality Controlled Biochemicals (QCB) as previously described [25]. The following antibodies were obtained from Cell Signaling Technology: GAPDH, phospho-TrkA, TrkA, phospho-ERK, ERK, phospho-JNK, JNK, phospho-ERK5, p27^Kip1^, PCNA (PC10), p21^Waf1/Cip1^. Anti-Rabaptin-5, anti-SYNGAP and anti-NMDAR2B antibodies were obtained from BD Biosciences, ABR Affinity Bioreagents and ZYMED, respectively.

### Generation of stable RabGEF1 antisense (AS) transfectants

The construction of the RabGEF1 AS expression plasmid has previously been reported by our laboratory [25]. Specifically, the mouse RabGEF1 cDNA clone was digested with XbaI and XhoI, and the entire full-length cDNA insert released was ligated to the pBK-CMV expression vector to generate the RabGEF1 antisense expression plasmid (RabGEF1-AS). The AS expression construct and the control pBK-CMV vector plasmid were transfected separately into PC12 cells using LIPOFECTAMINE Reagent (Life Technologies) according to the manufacturer’s instructions. Transfected cells were incubated in DMEM/20% HS/10% FCS for 72 hours and passaged at 1:10 dilution followed by addition of G418 at 600 μg/ml. After 4 weeks of selection, discrete resistant colonies were isolated and cultured further in medium with 400 μg/ml of G418. Positive clones were identified for reduced RabGEF1 protein expression using Western blot analysis with anti-RabGEF1 antibody.

### Quantification of neurite outgrowth

PC12 transfectants cultured for microscopy and neurite outgrowth measurement experiments were seeded in Lab-Tek II chamber slides (Nunc). At designated time points after NGF stimulation (50 ng/ml), images were captured with an Olympus BX60 microscope using a Retiga-2000R QImaging camera run by Image-Pro Plus (version 6.3) software (Media Cybernetics). To quantify neurite outgrowth, length of the longest neurite and total neurite length were measured. The total number of tip ends was also counted to represent the number of neurites derived from single individual cells [37].

### Immunoprecipitation for mass spectrometry and Western blot analysis

PC12 transfectant cells were lysed in 0.5% Triton-X in phosphorylation solubilization buffer (PSB: 50mM HEPES, 100mM NaF, 10mM Na_4_P_2_O_7_, 2mM Na_3_VO_4_, 2mM EDTA, 2mM NaMoO_4_.2H_2_O, pH = 7.35) containing protease inhibitors (Complete mini protease inhibitor cocktail tablets, Roche) for 60 min at 4°C. Following centrifugation (5 min, 16,000 X g), supernatants were incubated with 4 μl of affinity-purified rabbit anti-RabGEF1 polyclonal antibody (Quality Controlled Biochemicals; Hopkinton, MA) for 60 min at 4°C, then added to 20 μl Protein A/G PLUS agarose beads (Santa Cruz Biotech) and incubated for 16 hours at 4°C. The beads were washed 3 times in PBS and analyzed by either mass spectrometry or Western blotting.

### Mass spectrometry analysis

The immunoprecipitated proteins bound to the beads were analyzed by mass spectrometry by the Stanford University Proteomics and Integrative Research Facility. To identify interacting protein complexes, in-solution digests of the bound protiens underwent peptide separation by liquid chromatography. The peptides were fragmented in the mass spectrometer and a high capacity ion trap with high sensitivity capabilities for ion masses was used to identify peptides derived from proteins present in the purified complex. All analyses were conducted using a matrix-assisted laser desorption/ionization-time of flight mass spectrometry (MALDI-TOF MS) from Bruker Daltonics.

### Western blot analysis

Cells were lysed in Pierce IP Lysis Buffer (Pierce) supplemented with Halt Protease Inhibitor Cocktail (Thermo Scientific) or Complete mini protease inhibitor cocktail tablets (Roche), and the resulting lysates were separated by 10% SDS-PAGE and then electroblotted onto Invitrolon PVDF membranes (Novex, Life Technologies). Membranes were blocked in 5% nonfat dry milk in Tris-buffered saline-Tween 20 (0.1%) buffer and then probed with primary antibody in 5% BSA-Tris-buffered saline-Tween 20 (0.1%) buffer. The antigen-antibody complexes were visualized using horseradish peroxidase-conjugated secondary antibody to rabbit or mouse IgG (Cell Signaling Technology) with SuperSignal West Pico Chemiluminescent Substrate Kit (Thermo Scientific) and the images were captured by exposing membranes to autoradiography films (GeneMate). The films were scanned and specific signals were quantified by UN-SCAN-IT gel Version 5.3 (Silk Scientific).

### Activation of Ras, Rac1, and Cdc42

Ras activation was assayed using the EZ-Detect Ras Activation Kit (Pierce). A glutathione S-transferase (GST) protein containing the Ras-binding domain of Raf-1 was used to precipitate active GTP-bound Ras. Activation of Rac1 and Cdc42 was assayed using the EZ-Detect Rac1 Activation Kit (Pierce) or the EZ-Detect Cdc42 Activation Kit (Pierce), respectively. A GST fusion protein containing the p21-binding domain human Pak1 was used to precipitate active GTP-bound Rac1 or Cdc42.

### TrkA Internalization

CMV and AS transfectants were labeled at 4°C with biotinylated anti-rat TrkA antibody (R&D Systems), placed at 37°C for 0, 2.5, 15, 30 or 60 min, and incubated with APC-conjugated streptavidin (BD PharMingen) for 20 min on ice. Levels of surface TrkA were analyzed by flow cytometry.

### Flow cytometry

CMV and AS transfectants were stained with the indicated antibodies and analyzed on a FACSCalibur flow cytometer (BD Biosciences). Data were analyzed using Flow Jo software (Treestar).

### Nitric Oxide Synthase (NOS) Activity Assay

Activation of NOS activity by NMDA in PC12 cell transfectants was assessed using the NOS Activity Assay Kit (Cayman Chemical) according to the manufacturer’s instructions. Specifically, 10 μl of proteins extracted from each PC12 cell transfectant sample were incubated with 25 μl of 2X Reaction Buffer, 5 μl of 10 mM NADPH freshly prepared in 10 mM Tris-HCl (pH 7.4), 1 μl of L-[2,3,4,5-^3^H]arginine monohydrochloride (1 μCi/μl) (Amersham), 5 μl of 6 mM CaCl_2_ and 4 μl of H_2_O at room temperature for 60 min. The reaction was stopped by adding 400 μl of Stop Buffer and 100 μl of equilibrated resin. The reaction sample was transferred to a spin cup and centrifuged for 30 sec. The eluate was transferred to a scintillation vial and the radioactivity was quantitated using a liquid scintillation counter.

## Results

### Expression of RabGEF1 protein in PC12 cells stimulated with NGF and its suppression by antisense expression of RabGEF1 cDNA

**Fig 1:**
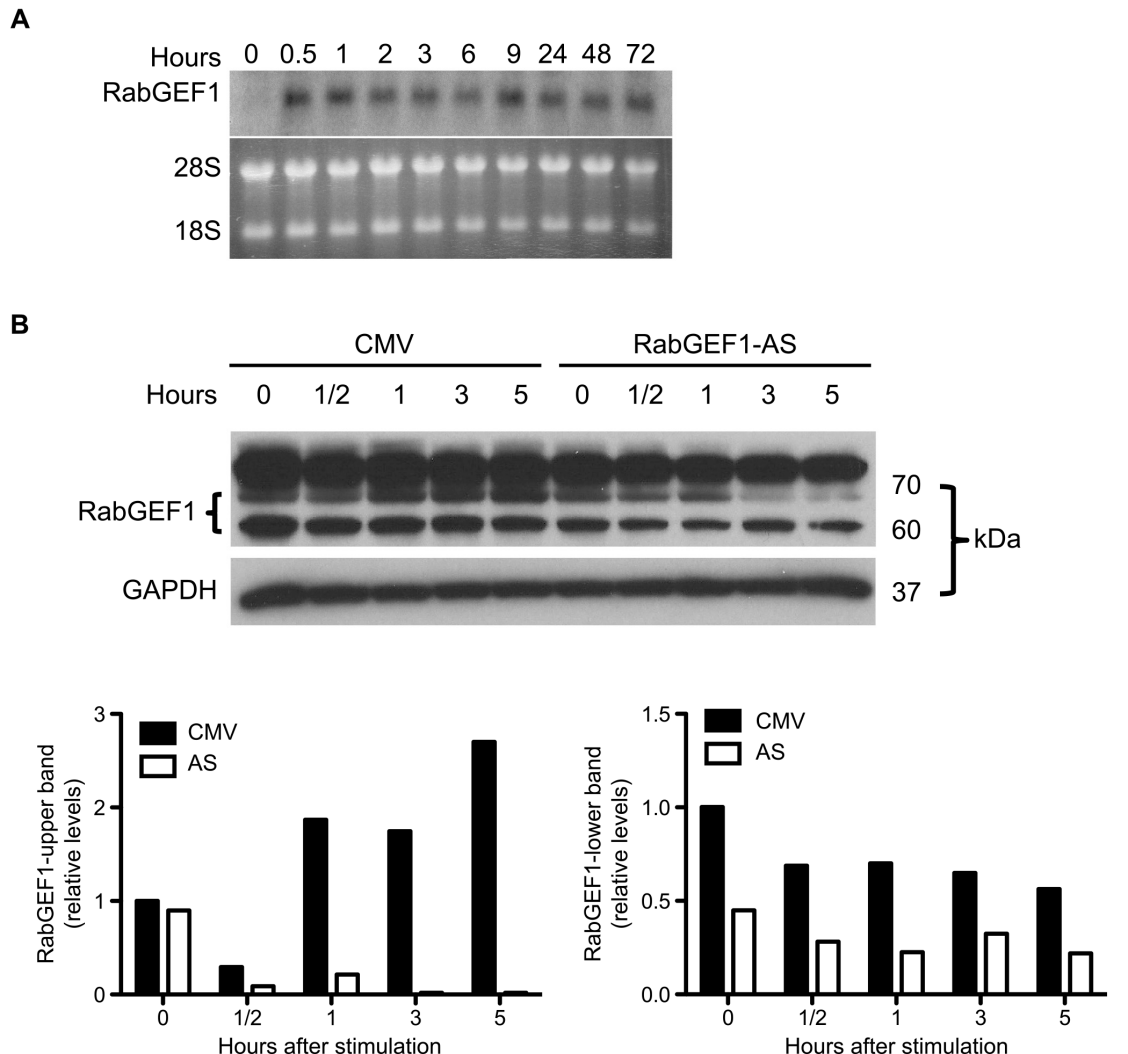
Expression of RabGEF1 in PC12 cells stimulated with NGF. A) Analysis of RabGEF1 mRNA expression in PC12 cells stimulated with NGF. PC12 cells were stimulated with NGF (50 ng/ml) for the indicated time periods and RNA was extracted and analyzed using Northern blot analysis. The bottom panel shows equal loading of RNA in each sample. B) Analysis of RabGEF1 protein expression in PC12 cells transfected with empty CMV vector or construct expressing antisense (AS) RabGEF1 mRNA. Stable PC12 transfectants expressing CMV or RabGEF1-AS construct were stimulated with NGF (50 ng/ml). Cells were lysed and RabGEF1 expression was assessed by Western blot analysis. The bottom panel shows equal loading of protein by probing the blots with anti-GAPDH antibody. Blot images were scanned and specific signals were quantified by UN-SCAN-IT gel Version 5.3. Data shown are representative of four independent experiments.

Using Northern blot and Western blot analyses, we have previously found that RabGEF1 mRNA and protein were expressed in high levels in the brain, suggesting that RabGEF1 may play an important role in the development and function of the central nervous system [25]. To support such a hypothesis, we first examined the expression of RabGEF1 mRNA in PC12 cells that had undergone neuronal differentiation in response to NGF stimulation. NGF induced a prolonged and sustained activation of RabGEF1 mRNA expression at all time points examined, including the 72-hour (3 days) time point, the longest time point we had examined (Fig 1A). This sustained pattern of mRNA activation was distinct from the transient pattern of expression observed with mouse mast cells stimulated through the IgE receptors or c-kit tyrosine kinase receptors [25,27], suggesting that RabGEF1 may also play a critical role in the differentiation and the maintenance of the differentiated state of PC12 cells by NGF.

Based on this pattern of prolonged activation of RabGEF1 mRNA expression, we reasoned that the generation of stable transfectants of PC12 cells expressing a steady level of reduced expression of RabGEF1 protein would offer a more useful approach to investigate the role of RabGEF1 in NGF-induced PC12 cell differentiation, which usually takes 7 days to fully complete. Moreover, stable transfectant lines can provide other advantages which transient transfection studies can not offer, such as a more uniform and sustained down-regulated expression of RabGEF1 and the large numbers of transfectant cells that can be generated in cultures, which are required for large scale studies such as signal transduction experiments. Our initial studies using three different RabGEF1-shRNA expression vectors targeting three distinct regions of the rat RabGEF1 mRNA found that the stable shRNA transfection approach significantly reduced the basal constitutive levels of RabGEF1 protein in the unstimulated transfected PC12 cells by >80% and therefore could potentially affect the growth, development and function of these stably transfected cells in cultures. Indeed, we found that all individual lines of PC12 cell transfectants obtained after antibiotic selection and expressing one of the three different RabGEF1-shRNA expression constructs failed to attain confluence even after they were cultured in coated tissue culture plates for a prolonged period of time. Moreover, extensive and rapid cell death occurred in these cultures once these cells had grown into large discrete colonies. On the other hand, stable PC12 transfectants expressing the empty shRNA vector or scrambled shRNAs were similar to untransfected PC12 cells in terms of their growth and ability to attain confluence in coated tissue culture dishes. These observations suggest that a threshold level of RabGEF1 expression in basal condition is critical for the growth of PC12 cells cultured in normal maintaining conditions.

We previously used the stable antisense (AS) cDNA expression approach in knocking down RabGEF1 protein expression in a mouse mast cell line, and the distinct advantage of using such approach was represented by our previous observation that overexpression of RabGEF1-AS mRNA did not appear to significantly affect the basal RabGEF1 protein expression in these cells, but instead inhibited the replenishment of RabGEF1 protein in the time periods subsequent to the onset of cell activation [25]. Indeed, our findings are consistent with those obtained in recent studies indicating that AS expression can inhibit sense gene expression in ways that are distinct from those regulated by shRNA expression [38,39].

Since the coding region of the mouse RabGEF1 cDNA is 96% homologous to the rat counterpart, we transfected PC12 cells with the mouse RabGEF1-AS expression construct that had previously been shown to be highly effective in knocking down RabGEF1 protein levels in mouse mast cells [25]. Stable lines of transfectants expressing the AS construct (PC12/RabGEF1-AS) or the empty CMV vector (PC12-/CMV) were established by selection in G418 and screened by assessing RabGEF1 protein levels in PC12 stimulated by NGF (50 ng/ml). Western blot analysis of rat RabGEF1 protein in PC12 cells revealed two distinct isoforms of molecular weights around 60–70 Kd (Fig 1B). Levels of the lower molecular weight RabGEF1 isoform (~60 Kd) in the AS transfectants were significantly lower (~50% reduction) than those observed in CMV transfectants at all time points examined. On the other hand, levels of the higher molecular weight RabGEF1 isoform (~70 Kd) in the control CMV transfectants were increased at 1, 3, and 5 hours after NGF stimulation. Basal unstimulated levels of the high molecular isoform in AS transfectants appeared to be similar to those in CMV transfectants, but these levels were significantly reduced at 3 and 5 hours after NGF stimulation, suggesting our AS expression approach was effective in knocking down the expression of certain intracellular pools of RabGEF1 protein in PC12 cells under conditions when these cells were specifically stimulated by NGF (Fig 1B). Four individual lines of both CMV and AS stable transfectants generated from two independent transfection studies were selected for further experiments, which showed similar results in all our studies. Representative data generated from one of each CMV and AS transfected clones are shown in the following sections.

### Antisense knockdown of RabGEF1 expression enhances cell proliferation and alters cell cycle progression

**Fig 2:**
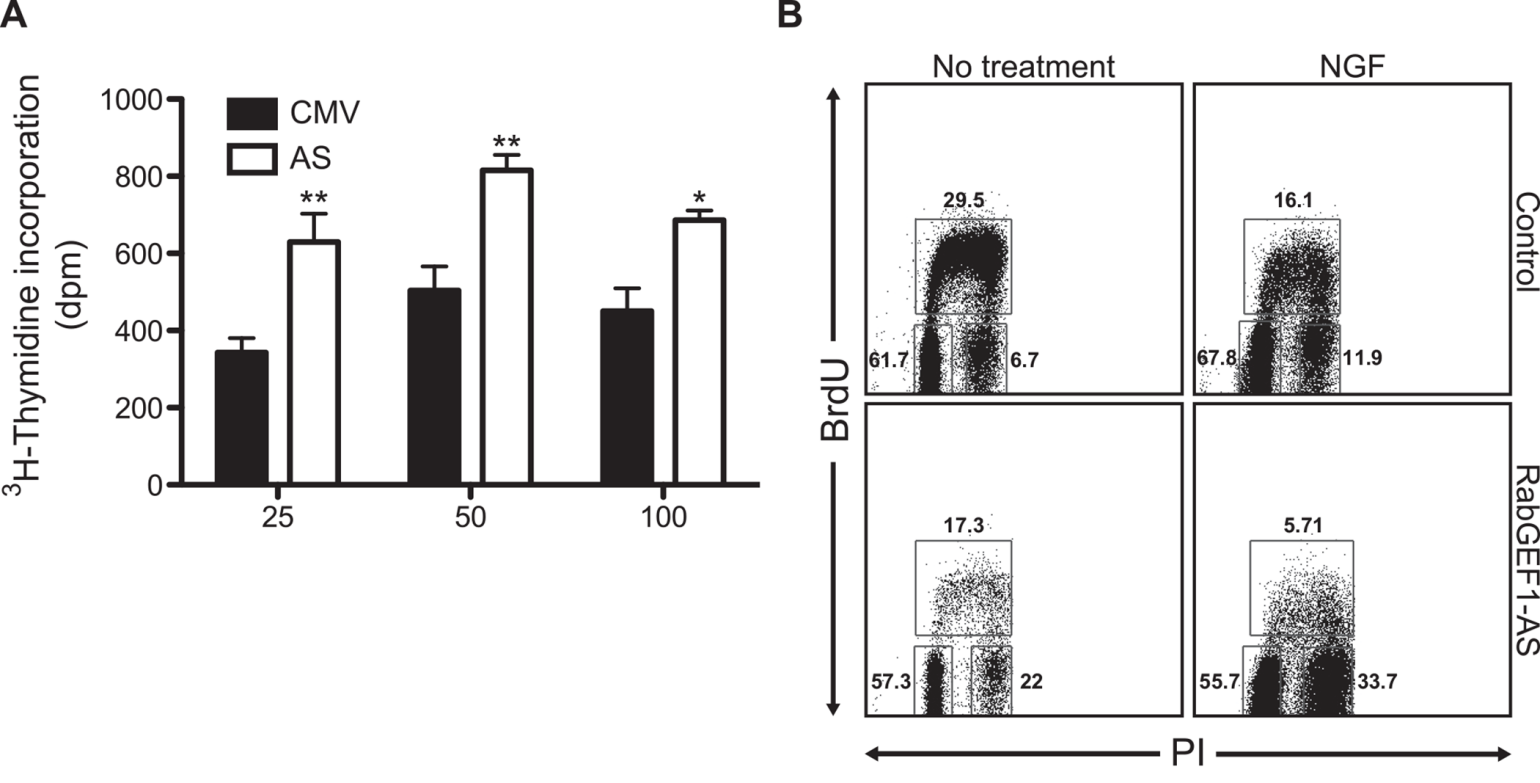
Reduced RabGEF1 expression enhances cell proliferation and altered cell cycle progression in NGF-differentiated PC12 cells. A) Enhanced cell proliferation in PC12 AS transfectants stimulated by NGF. PC12 transfectants were cultured overnight in 0.5% FCS and then incubated with NGF for 24 hours. Cells were then pulsed with 10 μCi/ml of ^3^H-thymidine for 4 hours and ^3^H-thymidine incorporation was counted. n = 6 for both CMV and AS samples. * p<0.05 by unpaired *t*-test, ** p<0.01 by unpaired *t*-test. B) Flow cytometric analysis of BrdU-labeled PC12 transfectants. CMV and AS transfectants were treated with NGF (50 ng/ml) for 6 days. Cells were then pulsed with 10 μM BrdU for 1 hour and then stained with anti-BrdU-FITC and PI. Samples were analyzed by flow cytometry. Data shown are representative of three independent experiments.

**Fig 3:**
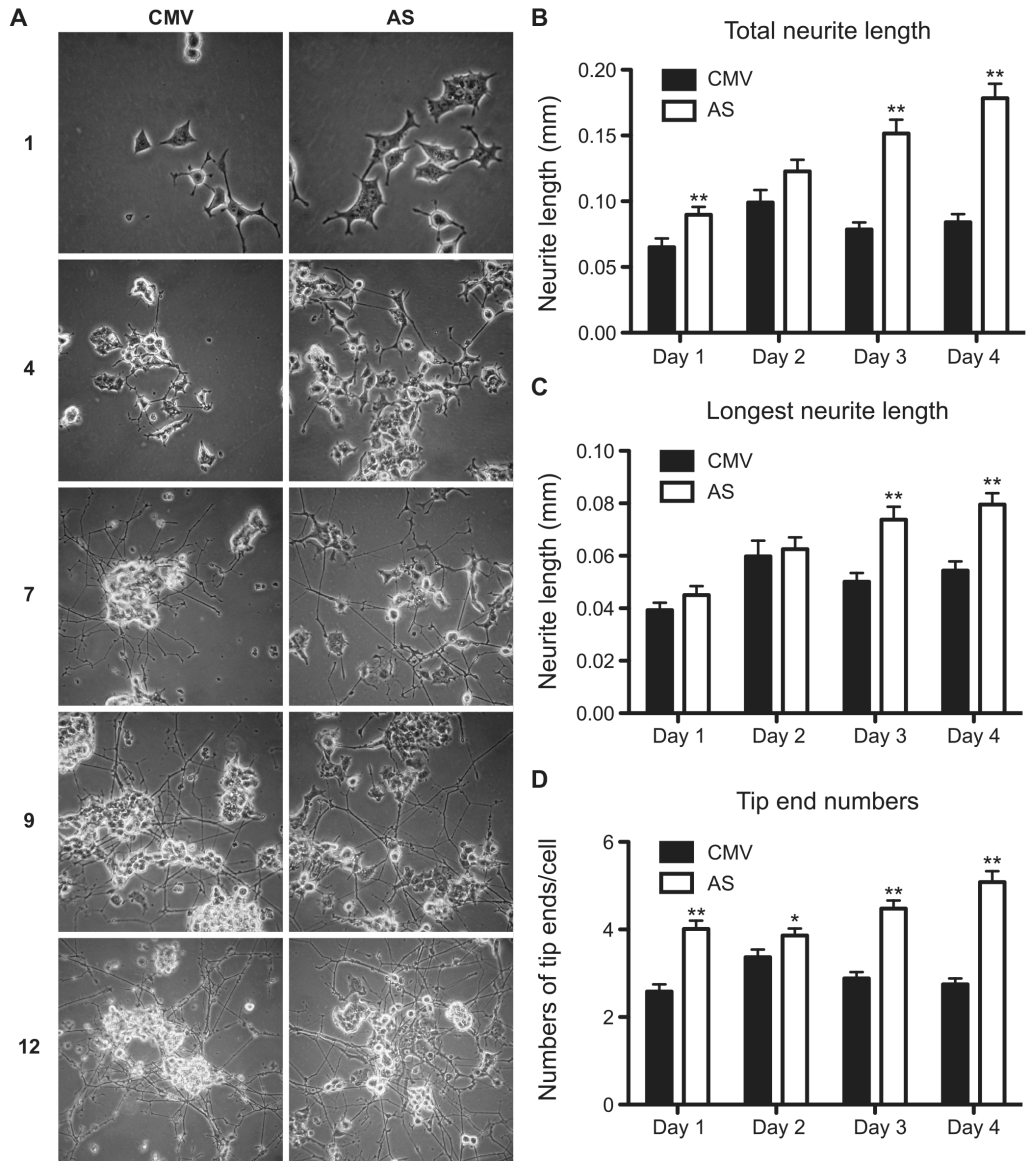
Reduced RabGEF1 expression enhances neurite outgrowth in NGF-differentiated PC12 cells. A) Phase-contrast photomicrographs showing morphological characteristics of PC12 cells transfected with RabGEF1-AS construct or control empty CMV vector. PC12-CMV control and PC12-AS transfectants were grown on collagen-coated plates and stimulated with NGF (50 ng/ml) for 1, 4, 7, 9, or 12 days. B)—D) AS transfectants undergoing differentiation induced by NGF exhibited increased total neurite length, longest neurite length, and tip end numbers at 1 day (CMV: n = 84; AS: n = 73), 2 days (CMV: n = 87; AS: n = 96), 3 days (CMV: n = 113; AS: n = 84) or 4 days (CMV: n = 103; AS: n = 86) after stimulation, where n = number of cells counted. Data shown were pooled from three separate experiments. * p<0.05 by unpaired *t*-test, ** p<0.01 by unpaired *t*-test.

AS transfectants and CMV controls were stimulated with NGF (50 ng/ml) and the phenotypic changes associated with NGF stimulation were assessed. We found that the AS transfectants exhibited a higher rate of cell proliferation in response to NGF treatment compared to that of CMV controls, suggesting that these cells were less affected by the growth arrest effect exerted by NGF. As shown in Figs 2A and 3A, AS transfectants appeared to continue proliferating into large colonies after NGF treatment, whereas CMV control cells appeared to stop proliferating after NGF stimulation. However, AS transfection did not appear to alter the cell proliferation rate in the basal unstimulated state (CMV: 1039 ± 81 cpm; AS: 992 ± 115 cpm; p = 0.745, n = 6). These data suggest that RabGEF1 can regulate neuronal differentiation by inhibiting cell proliferation after NGF stimulation. Previous studies have shown that PC12 cells treated with NGF for 2 weeks are blocked in G1 phase of the cell cycle [40]. In our studies, cell cycle analysis of the BrdU-labeled PC12 tranfectants showed that AS transfectants were arrested at the G2/M phase when cultured in either unstimulated or NGF-treated conditions (**-NGF**: CMV: 6.7% vs. AS: 22.0%; **+NGF**: CMV: 11.9% vs. AS: 33.7%) (Fig 2B). These data thus suggest that RabGEF1 is involved in the cell cycle progression in PC12 cells undergoing neuronal differentiation.

### Antisense knockdown of RabGEF1 expression enhances neurite outgrowth induced by NGF

Previous studies have shown that Rab5 or RabGEF1 expression can regulate NGF-induced neurite outgrowth in PC12 cells [12]. Thus, we assessed whether our AS transfectants exhibited different patterns and kinetics of neurite outgrowth compared to the CMV controls. Morphological analysis of AS transfectants that were stimulated by NGF for 1, 4, 7, 9 or 12 days showed that the induction of neurite outgrowth by NGF occurred earlier in these cells compared to CMV control cells (Fig 3A). Moreover, we found that all three parameters representing neurite outgrowth, such as total neurite length, length of the longest neurite, and tip end numbers (numbers of neurites generated from individual cells), were significantly enhanced in AS transfectants that were stimulated by NGF for 1, 2, 3 or 4 days (Fig 3B–3D). Our observations are thus consistent with the previous findings that Rab5 or RabGEF1 can negatively regulate NGF-induced neurite outgrowth in PC12 cells [12].

### Antisense knockdown of RabGEF1 expression reduces NGF-induced TrkA phosphorylation but not its internalization

**Fig 4:**
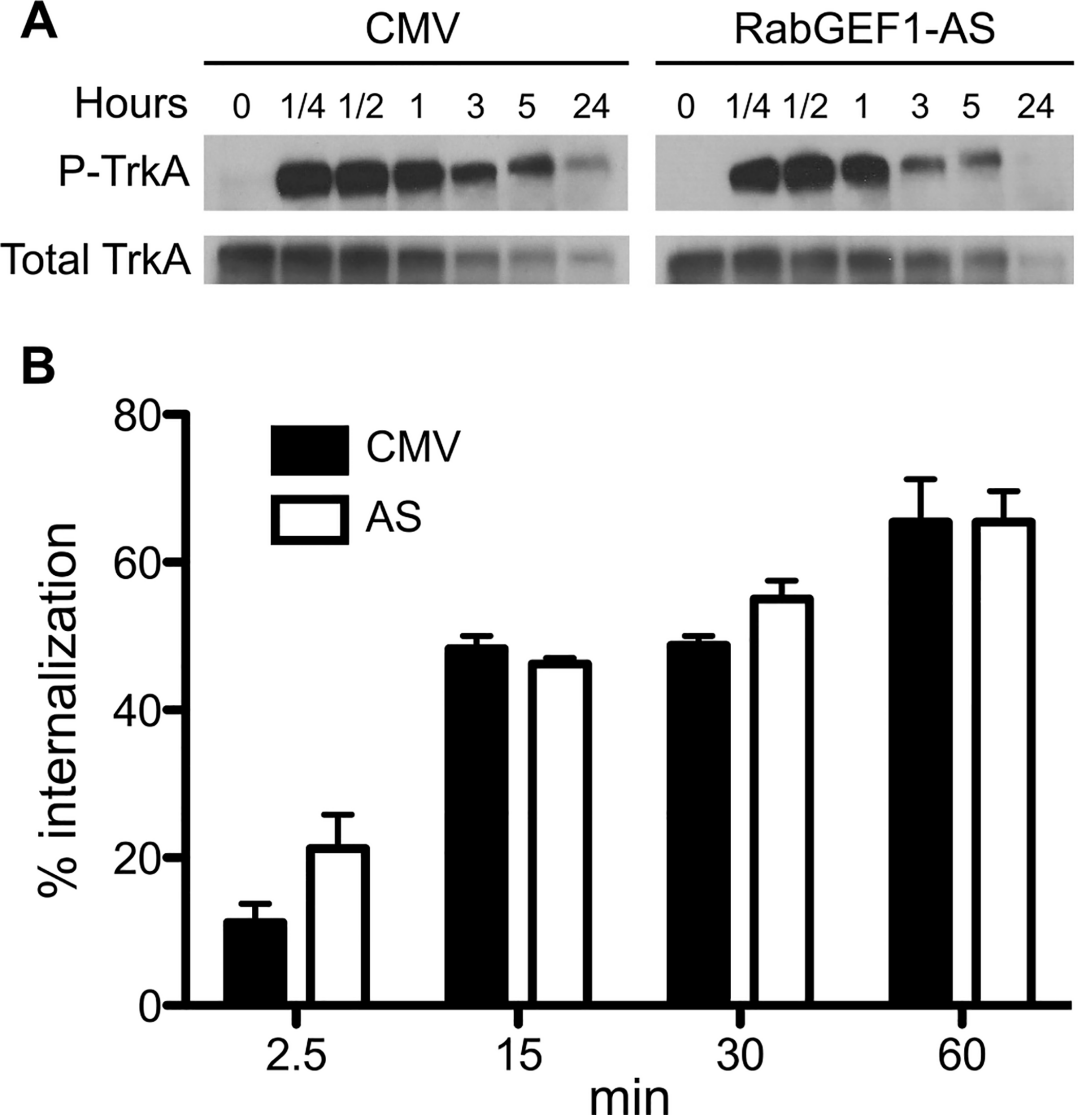
Reduced RabGEF1 expression decreases NGF-induced TrkA phosphorylation but not TrkA internalization. A) CMV and AS stable transfectants were stimulated by NGF (50 ng/ml) for 30 min, 1 hour, 3 hours, or 5 hours. Total cell lysates were analyzed by Western blot using anti-phospho-TrkA and anti-TrkA antibody. B) CMV and AS stable transfectants were incubated with biotinylated anti-rat TrkA IgG antibody for the indicated times. Surface TrkA was assessed by streptavidin (SA)-APC fluorescence and analyzed by flow cytometry. Percentage TrkA internalization was calculated by subtracting mean fluorescence intensity at 2.5 min, 15 min, 30 min, or 60 min from mean fluorescence intensity at 0 min and dividing this number by mean fluorescence intensity at 0 min (x 100%). Data shown were pooled from four separate experiments.

We next examined NGF-induced signal transduction pathways to elucidate the underlying mechanisms that mediated the distinct phenotypic responses we observed in AS transfectants. Stimulation of NGF induces the phosphorylation of TrkA receptors, which is followed by TrkA internalization and trafficking through the endocytic pathway [6]. We found that the levels of TrkA phosphorylation induced by NGF stimulation were reduced in AS transfectants compared to CMV control cells at 3, 5 and 24 hours after NGF treatment. However, total TrkA levels appeared not to be significantly affected by RabGEF1 antisense expression (Fig 4A). On the other hand, we have previously shown that RabGEF1can down-regulate IgE-dependent mast cell activation by promoting the internalization of FcεRI receptors after their crosslinking by IgE and antigen [26]. Thus we examined whether the internalization of TrkA receptors after their activation by NGF was regulated by RabGEF1 expression in PC12 cells. Previous studies have shown that anti-receptor antibodies can induce ligand-independent receptor activation for members of the receptor tyrosine kinase family [41,42]. Specifically, anti-rat TrkA IgG antibody has been shown to be as effective as NGF in activating TrkA and its downstream signaling pathways and the induction of neurite outgrowth [43]. Therefore, in this study, we used a biotinylated anti-rat TrkA IgG antibody to assess % internalization of TrkA after TrkA activation induced by the binding of the antibody to TrkA in both CMV and AS transfectants. As shown in Fig 4B, incubation with the anti-rat TrkA IgG antibody induced TrkA internalization in CMV transfectants in a time-dependent manner. A parallel internalization studies with AS transfectants showed that % internalization of TrkA receptors was similar between AS and CMV transfectants for all the time points chosen in this study (Fig 4B). Taken together, RabGEF1 expression appeared to positively regulate TrkA activation by NGF, but did not appear to influence TrkA internalization induced by TrkA activation.

### RabGEF1 positively regulates Ras activation in NGF-differentiated PC12 cells

**Fig 5:**
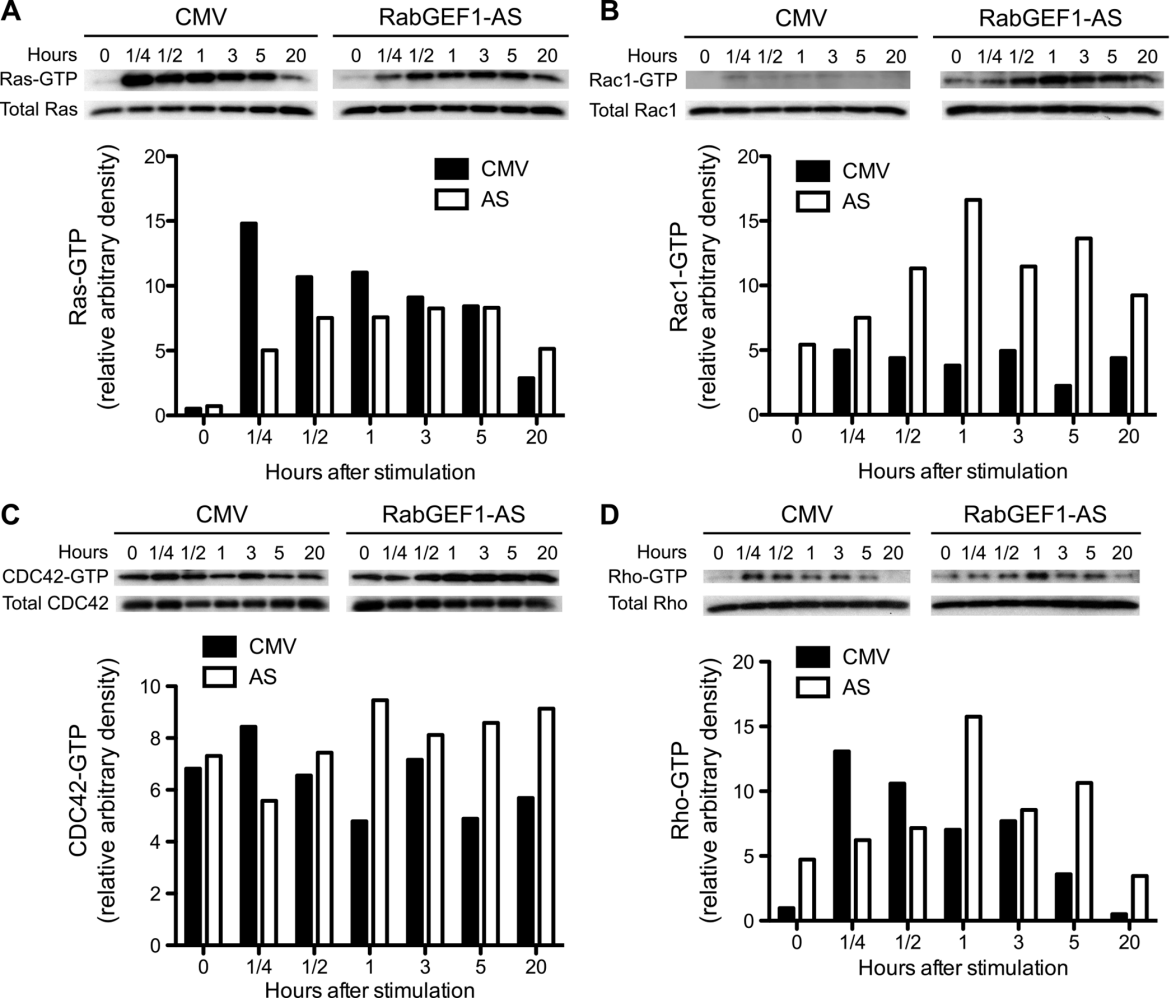
RabGEF1 differentially regulates activation of Ras, Rac1, and Cdc42 in NGF-differentiated PC12 cells. A) NGF-induced Ras activation was reduced in RabGEF1-AS transfectants. CMV and AS transfectants were stimulated by NGF (50 ng/ml) for the indicated times. Activated Ras-GTP was affinity-precipitated with GST fusion protein containing the Ras binding domain of Raf-1 and analyzed by Western blotting using anti-Ras antibody. B) Enhanced Rac1 activation in RabGEF1-AS transfectants stimulated by NGF (50 ng/ml). Activated Rac1-GTP was affinity-precipitated with GST fusion protein containing the p21-binding domain of Pak1 and analyzed by Western blotting using anti-Rac1 antibody. C) Enhanced Cdc42 activation in RabGEF1-AS tranfectants stimulated by NGF. Activated Cdc42-GTP was affinity-precipitated using GST fusion protein containing the p21-binding domain of Pak1 and analyzed by Western blotting using anti-Cdc42 antibody. D) Kinetics of Rho activation in PC 12 transfectants stimulated by NGF. Activated Rho-GTP was affinity-precipitated with GST fusion protein containing the Rho-binding domain of Rhotekin and analyzed by Western blotting using anti-Rho antibody. Blot images were scanned and specific signals were quantified by UN-SCAN-IT gel Version 5.3. Data shown are representative of three independent experiments.

Stimulation of PC12 cells by NGF leads to the activation of Ras, which mediates the induction of neurite outgrowth in these cells in response to NGF [3,4]. We previously showed that RabGEF1 binds to Ras to exert its negative regulatory effects on Ras/ERK signaling pathways in mouse mast cells [25]. Therefore, we examined whether reduced RabGEF1expression in AS transfectants was associated with a similar effect on Ras activation. Using a Ras-GTP “pull down” (affinity-precipitation) assay, we assessed the levels of Ras activation in PC12 transfectants after NGF stimulation. As illustrated in Fig 5A, AS transfectants exhibited reduced levels of Ras activation compared to those of CMV control cells at 15 min, 30 min and 1 hour after NGF stimulation, suggesting that RabGEF1 is a positive regulator of Ras activation in NGF-stimulated PC12 cells. This finding is in direct contrast to that obtained from our previous studies with mouse mast cells stimulated via the high-affinity IgE receptors or c-kit receptors [25,27], which have shown that RabGEF1 acts as a negative regulator of Ras activation.

### RabGEF1 negatively regulates activation of Rac1 and Cdc42 in NGF-differentiated PC12 cells

In addition to Ras activation, activation of Rho GTPase such as Rho, Rac1 and Cdc42, is induced in PC12 cells stimulated by NGF and is thought to mediate neurite outgrowth [19,20,22,44]. Using a GST “pull down” assay for Rac1-GTP or Cdc42-GTP, we assessed whether a potentiation of activation of Rac1 or Cdc42 was associated with the enhanced neurite outgrowth observed with the AS transfectants. We found that AS transfectants exhibited a significantly higher and more sustained activation of Rac1 activity and Cdc42 activity (Fig 5B and 5C). Moreover, the kinetics curves of activation of Rac1 and Cdc42 observed with AS cells appeared to shift to the right with the peak of GTPase activation occurring at longer time points of NGF stimulation (Fig 5B and 5C). These results suggest that RabGEF1 can act as a negative regulator of activation of both Rac1 and Cdc42. These findings are different from those obtained with mouse mast cells activated through high-affinity IgE receptors [25]. On the other hand, we did not detect any significant differences in levels of Rho activation between CMV and AS cells after NGF stimulation, although the time point for peak Rho activation appeared to be shifted from 15 min in control cells to 1 hour in AS transfectants (Fig 5D). These results suggest that Rho activation may not be directly involved in RabGEF1-mediated signaling responses in PC12 cells.

### RabGEF1 can bind to Ras, Rac1 and Cdc42 in PC12 cells

**Fig 6:**
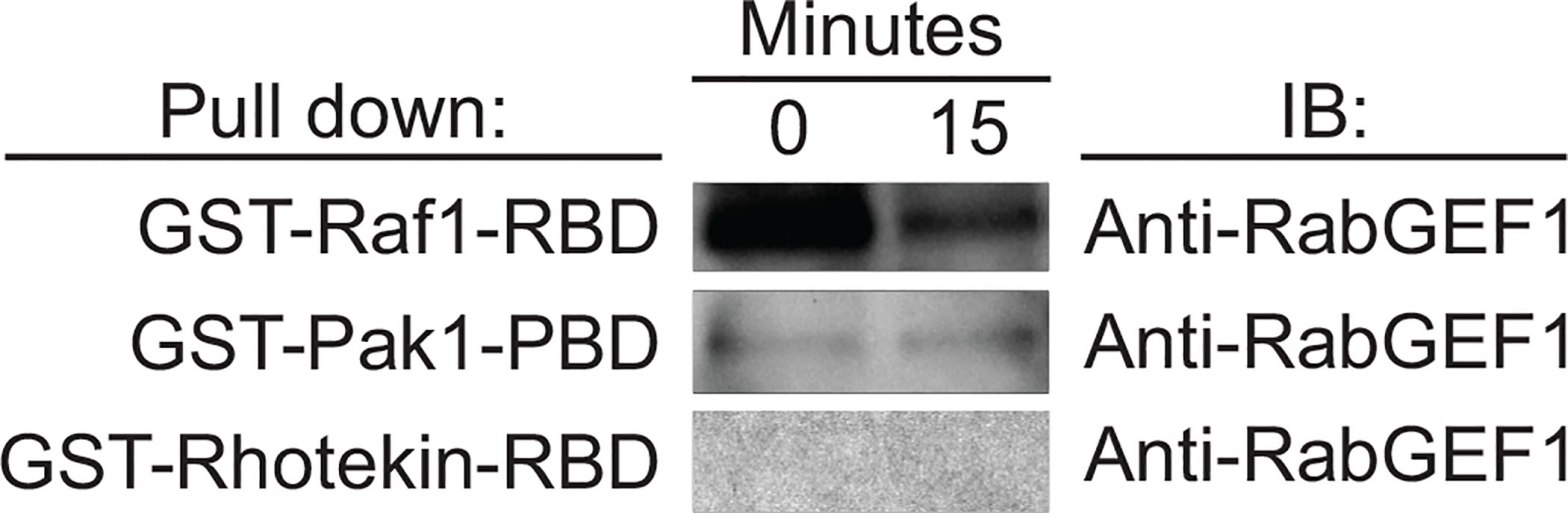
RabGEF1 binds directly to Ras, Rac1 and Cdc42. Binding of RabGEF1 to Ras and Rac1/Cdc42, but not Rho. Cell lysates derived from PC12 cells stimulated by NGF for 0 or 15 min were subjected to “pull down” (affinity-precipitation) assays using GST-Raf1-RBD fusion protein, GST-Pak1-PBD fusion protein, or GST-Rhotekin-RBD fusion protein. Proteins bound to the beads were analyzed by Western blotting using anti-RabGEF1 antibody. Data shown are representative of three independent experiments. IB: Immunoblotting.

We previously showed that RabGEF1 can bind to Ras to exert its negative regulatory effects on Ras/ERK signaling pathway in mast cells [25]. Thus, we assessed whether RabGEF1 also exerted its regulatory effects on the activation of Rac1 and Cdc42 through its direct association with Rac1 and Cdc42. Consistent with what we have observed with mast cells [25], GST-fusion protein containing the Raf1-RBD (Ras binding domain) was able to “pull down” active Ras-GTP and RabGEF1 protein from lysates derived from both stimulated and unstimulated PC12 cells (Figs 5A and 6). Similarly, we found that active Rac1-GTP and Cdc42-GTP, together with the RabGEF1 protein, were “pulled down” by GST-fusion protein containing the Pak1-PBD (p21 binding domain) from both stimulated and unstimulated PC12 cell lysates (Figs 5B, 5C and 6). However, GST-fusion protein containing the Rhotekin-RBD (Rho-binding domain) was able to “pull down” active Rho-GTP (Fig 5D), but not RabGEF1 protein from such lysates (Fig 6). These data thus suggest that RabGEF1 can bind to Ras, Rac1 and Cdc42, but not Rho, and such interactions may mediate the effects of RabGEF1 on the activation of these small GTPases by NGF stimulation in PC12 cells.

### RabGEF1 regulates TrkA-mediated downstream signaling pathways in NGF-differentiated PC12 cells

**Fig 7:**
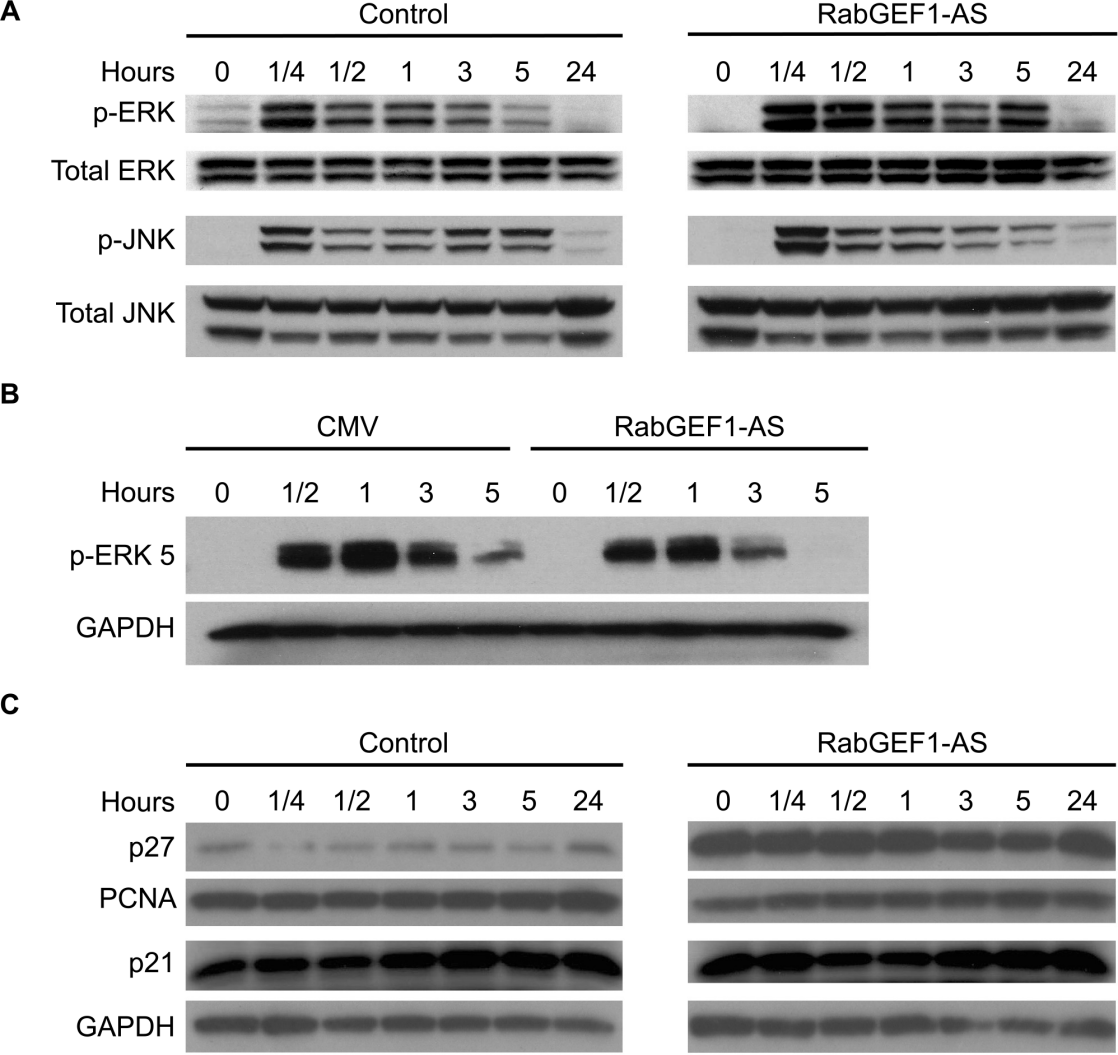
RabGEF1 regulates TrkA-mediated downstream signaling pathways in NGF-differentiated PC12 cells. A) Enhancement of ERK and JNK activation in PC12 cells transfected with RabGEF1-AS expression construct in response to NGF stimulation. Cell lysates were analyzed by Western blotting and probed with antibody against phospho-ERK or phospho-JNK. B) Reduced ERK5 activation in PC12 cells transfected with RabGEF1-AS expression construct in response to NGF stimulation. Cell lysates were analyzed by Western blotting and probed with antibody against phospho-ERK5. C) Western blot analysis of expression of p27^Kip1^, PCNA (PC10) and p21^Waf1/Cip1^ in CMV and RabGEF1-AS PC12 transfectants stimulated by NGF (50 ng/ml).

We next assessed whether the signaling pathways downstream of TrkA were regulated by RabGEF1 expression in NGF-differentiated PC12 cells. Since Ras activation was down-regulated in NGF-stimulated AS transfectants (Fig 5A), we assessed the activation of ERK/MAP kinase, which serves as the immediate downstream signaling response of Ras activation. Interestingly, contrary to what we found with Ras activation (Fig 5A), we observed a significant enhancement in levels of ERK/MAP kinase activation in the AS transfectants at time points ranging from 15 min to 5 hours after NGF treatments (Fig 7A). On the other hand, since activation of both Rac1 and Cdc42 was enhanced in NGF-stimulated AS transfectants, we examined the activation of JNK kinase, which is known to be induced by the activation of immediate upstream p21-activated kinase (PAK), which, in turn, acts as an effector of Rac1 and Cdc42. Consistent with our finding with enhanced activation of Rac1 and Cdc42, AS transfectants exhibited a potentiation of JNK kinase activation induced by NGF stimulation in PC12 cells (Fig 7A). Therefore, it appeared that the TrkA-mediated activation of Ras and Rac1 was modulated by RabGEF1 in opposite manners, whereas their downstream signaling responses were regulated by RabGEF1 expression in a similar fashion. These findings may be explained by the previous findings that Rac1 and Cdc42 can interact with Raf-1 in a synergistic manner to activate ERK via pathways that involve phosphorylation of both MEK1 by PAK1 and Raf-1 by PAK-3 [45,46]. In addition, the activation of Rac1-PAK signaling has been shown to stimulate ERK activation via the regulation of formation of MEK1-ERK complexes [47]. Moreover, a recent study has shown that Rac1 activation can lead to increased levels of ERK phosphorylation [48].

To further assess the role of RabGEF1 in the regulation of TrkA-dependent signaling events, we assessed whether ERK5 phosphorylation induced by NGF was modulated by RabGEF1 expression. ERK5 is a member of the MAPK superfamily that is required for neurite outgrowth in PC12 cells induced by NGF by a Ras-independent mechanism [49]. We found that levels of ERK5 phosphorylation induced by NGF stimulation were down-regulated in RabGEF1-AS transfectants (Fig 7B), suggesting that RabGEF1 can also regulate other signaling events that involve Ras-independent activation of MAPK. Taken together, these data suggest that RabGEF1 is a negative regulator of downstream signaling pathways activated by Rac1 activation, which has been shown to mediate the induction of neurite outgrowth in NGF-differentiated PC12 cells [22,44].

Since AS transfectants were found to be arrested a the G2/M phase of the cell cycle when cultured in either unstimulated or stimulated conditions (Fig 2B), we examined the signaling pathways that mediated such effects of RabGEF1 on cell cycle progression. Using a protein array that assessed changes in expression levels of 400 cell cycle-related proteins in NGF-stimulated AS and control cells, we generated a list of proteins whose expression levels were significantly affected by RabGEF1 expression in PC12 cell transfectants (S1 Table). Using Western blot analysis, we further confirmed that the expression of p27^kip1^, an inhibitor of cyclin/cyclin-dependent kinase complex, was up-regulated, whereas the expression of proliferating cell nuclear antigen (PCNA) was down-regulated, in AS transfectants compared to control CMV cells upon NGF stimulation (Fig 7C). However, the expression of p21^Waf1/Cip1^, another cyclin-dependent kinase inhibitor that regulates cell cycle progression at G1 and S phase, was not affected by reduced RabGEF1 expression in AS transfectants (Fig 7C). These data suggest that p27^kip1^ and PCNA may be involved in the regulatory effects of RabGEF1 on cell cycle progression in neuronal differentiation of PC12 cells.

### RabGEF1 can bind to NMDA receptor NR2B subunit and its associated binding partner SynGAP in PC12 cells

**Fig 8:**
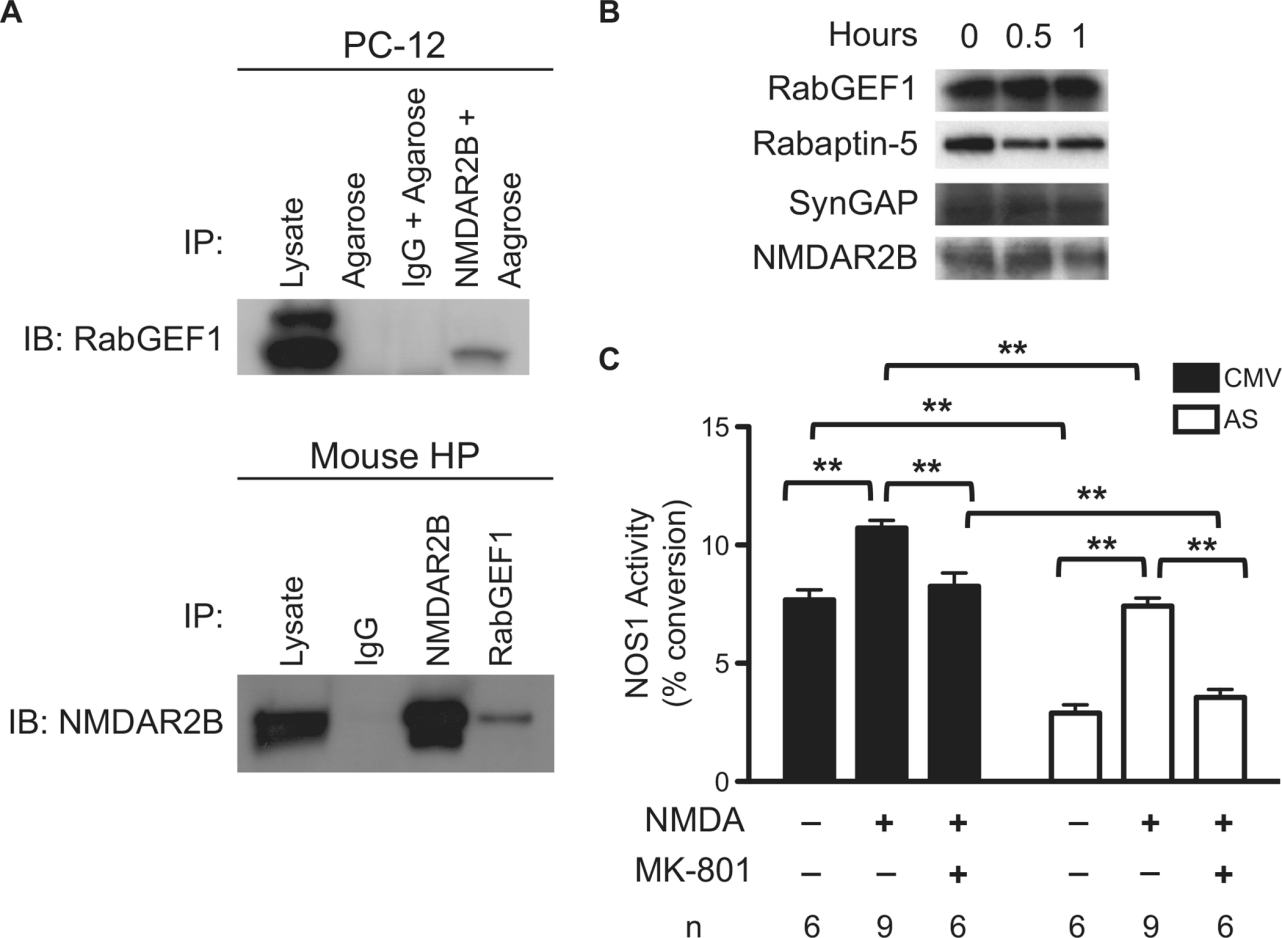
RabGEF1 binds to NMDA receptor subunit NR2B and negatively regulates NMDA-mediated NOS1 activation in NGF-differentiated PC12 cells. A) Binding of RabGEF1 to NMDA receptor NR2B subunit (NMDAR2B) in PC12 cells and mouse hippocampus (HP). Cell lysates were immunoprecipitated (IP) with anti-NR2B antibody or anti-RabGEF1 antibody and then immunoblotted (IB) with anti-RabGEF1 antibody or anti-NR2B antibody, respectively. Rabbit IgG was used as the negative control and the positive control was represented by total cell lysate. B) Binding of RabGEF1 to SynGAP in PC12 cells stimulated by NGF for 0, 0.5 or 1.0 hour. Cell lysates were immunoprecipitated with anti-RabGEF1 antibody and immunoblotted with anti-SynGAP antibody, anti-Rabaptin-5, anti-NR2B antibody, or anti-RabGEF1 antibody, respectively. C) Activation of NOS1 activity following NMDA receptor stimulation in PC12 cells transfected with CMV or RabGEF1-AS expression construct. Transfectants were differentiated by NGF stimulation (50 ng/ml) for 3 days and then stimulated with NMDA (750 μM) for 10 min. Production of ^3^H-citrulline was assayed using ^3^H-arginine as the substrate, and NOS1 activity was calculated as % citrulline conversion in the reaction in relation to total counts. Data shown were pooled from three separate independent experiments (n = number of samples). ** p<0.01 by unpaired *t*-test.

The differential effects of RabGEF1 on the activation of Ras, Rac1 and Cdc42 in PC12 cells suggest that RabGEF1 can interact with other regulatory factors in a PC12 cell-specific manner to elicit its distinct effects on neuronal differentiation in these cells. To identify RabGEF1-interacting proteins in NGF-stimulated PC12 cells, we used mass spectrometry to analyze proteins that were associated with RabGEF1 and immunoprecipitated by anti-RabGEF1 antibody. The most relevant positive “hits” were found to be represented by more than 100 proteins (S2 Table). However, several proteins, including the NMDA receptor NR2B subunit and its associated protein SynGAP, were consistently retrieved multiple times from the mass spectrometry analysis of a single sample or samples treated with different stimulation conditions (S2 Table). To confirm whether NR2B and SynGAP were indeed RabGEF1 bindings partners, PC12 or mouse hippocampus cell lysates were immunoprecipitated with specific anti-RabGEF1 or anti-NR2B antibody. Western blot analyses showed that RabGEF1 was immunoprecipitated together with NR2B and SynGAP by the anti-RabGEF1 or anti-NR2B antibody (Fig 8A and 8B). The specificity of the immunoprecipitation was further verified by the co-immunopreciptation of Rabaptin-5 [24], a known RabGEF1 binding partner, in the same precipitated complex (Fig 8B). Together, these data suggest that RabGEF1 can bind to NMDA receptor NR2B subunit and its associated binding partner, SynGAP.

### RabGEF1 negatively regulates NMDA-mediated functional responses in NGF-differentiated PC12 cells

Previous studies have shown that nitric oxide (NO) and neuronal NO synthase isoform NOS1 are important regulatory factors required for NGF-induced PC12 neuronal differentiation [50]. NGF-differentiated PC12 cells express both NOS 1 and NMDA receptors, including the NR2B subunit [50]. These differentiated cells can be activated by NMDA receptor stimulation, resulting in the activation of NOS1 and the production of NO [50]. Therefore, we compared the effects of NMDA stimulation on the activation of NOS1 in NGF-treated AS transfectants and CMV controls to assess whether RabGEF1 regulated NMDA-mediated functional responses in NGF-differentiated PC12 cells. AS and CMV transfectants were treated with 50 ng/ml NGF for 3 days, and the differentiated cells were subsequently stimulated with 750 μM NMDA for 10 min and then harvested for the NOS1 activity assay. NMDA stimulation induced significant increases in NOS1 activity in both transfectants (Fig 8C). Notably, the basal unstimulated NOS1 activity of AS cells was significantly lower (-62%) than that of CMV controls (p<0.01). However, NMDA stimulation was found to induce higher folds of increase in NOS1 activity in AS cells (+2.6 folds) compared to those of CMV controls (+1.4 fold) (p<0.01). Furthermore, pretreatment with MK-801, a selective NMDA receptor antagonist, significantly reversed NMDA-induced NOS1 activity in both AS and CMV cells (Fig 8C). These results indicate that RabGEF1 can positively regulate basal NOS1 activity but negatively regulate NMDA receptor-meditated NOS1 activation in NGF-differentiated PC12 cells, suggesting that RabGEF1 can interact with NMDA receptor-dependent signaling pathways to regulate cellular activation processes.

## Discussion

PC12 cells stimulated by NGF undergo neuronal differentiation with the induction of neurite outgrowth [5]. The NGF-TrkA complex formed by the binding of NGF to TrkA receptors is endocytosed into the cells and resides in the signaling endosomes [6]. Rab5 is expressed in these signaling endosomes, which are involved in the retrograde transport of NGF along the axons to the cell bodies [7]. Recent reports have shown that members of the Rab5 subfamily, such as Rab5, Rab21 and Rab22, can play significant roles in the regulation of neurite outgrowth in PC12 cells that undergo NGF-induced neuronal differentiation. Overexpression studies have suggested that Rab5 and Rab21 promote the early endosomal pathway destined for late endosomes/lysosomes and degradation, which, in turn, reduces NGF signaling and its associated neurite outgrowth [35]. On the other hand, Rab22 has been found to be a positive regulator of NGF-dependent signaling and neurite outgrowth in PC12 cells [35]. These data suggest that different members of the Rab5 subfamily of small GTPases can regulate distinct cellular processes in the signaling endosomal pathways to regulate neurite outgrowth in neuronal differentiation.

RabGEF1 (also known as Rabex-5), a guanine nucleotide exchange factor that activates Rab5, has also recently been shown to be a Rab22 effector [51]. Rab22-GTP acts as the early endosomal binding site for RabGEF1 and thereby recruiting RabGEF1 to the early endosomes [51]. However, there have been conflicting data regarding the role of RabGEF1 in the NGF-induced neuronal differentiation of PC12 cells. In an earlier study, constitutive activation of Rab5 or transient overexpression of RabGEF1 has been shown to block NGF-induced neurite outgrowth in PC12 cells [12]. Our data presented in this study are thus consistent with those obtained in that particular study, which implicate RabGEF1 as a negative regulator of neurite outgrowth. However, a recent report has shown that transient expression of a RabGEF1-shRNA can inhibit neurite outgrowth in the transfected NGF-stimulated PC12 cells, suggesting that RabGEF1 can function as a positive regulator of neurite outgrowth [35]. In our present study, we used the stable AS expression approach to induce a sustained knock-downed RabGEF1 expression in PC12 cells. We showed that levels of some intracellular pools of RabGEF1 protein were lowered by AS expression in the basal unstimulated condition, whereas levels of the other pools of RabGEF1 were reduced when the cells were specifically stimulated by NGF (Fig 1B). Under such conditions, reduced RabGEF1 expression was found to significantly enhance neurite outgrowth in NGF-treated PC12 cells, suggesting that RabGEF1 is a negative regulator of neurite outgrowth (Fig 3B–3D). Our studies thus suggest that data generated for functional phenotypic studies using the AS expression approach can be distinct from those obtained using the shRNA approach, since these two different approaches modulate the patterns of gene expression differently by activating distinct cellular pathways and mechanisms. Recent studies have suggested that AS expression can regulate threshold dependent gene expression by preferentially suppressing sense expression in the low range, but such inhibition is reduced when the level of sense expression becomes high [38,39].

On the other hand, we found that the levels of phospho-TrkA were reduced in AS tranfectants after NGF stimulation even though the total TrkA levels appeared to remain the same in these cells (Fig 4A). Furthermore, the levels of TrkA internalization appeared to be similar between AS tranfectants and CMV controls after NGF stimulation (Fig 4B). Thus a negative regulation of the levels of dephosphorylation of TrkA after its internalization appears to be one of the roles of RabGEF1 in regulating NGF-induced neuronal differentiation in PC12 cells. This interpretation is indeed consistent with our finding that levels of Ras activation were reduced in AS tranfectants compared to those observed with controls after NGF stimulation (Fig 5A).

Using the stable AS transfectant cell lines that were generated in this study, we were able to further assess other cellular processes that were influenced by the sustained reduction of RabGEF1 expression in NGF-differentiated PC12 cells. Our data suggest that RabGEF1 can play a significant role in the regulation of growth arrest and cell cycle progression when the cells are undergoing differentiation induced by a growth factor such as NGF. Our studies showed that PC12 cells lacking RabGEF1 expression exhibit higher rate of proliferation in response to NGF stimulation and that these cells are arrested at the G2/M phase in both basal and stimulated conditions (Fig 2A and 2B). These changes were accompanied by an increased expression of p27^kip1^ and a decreased expression of PCNA, suggesting RabGEF1 can regulate cell cycle progression by regulating the expression of important factors that modulate cell cycles (Fig 7). On the other hand, there are abundant evidence to implicate the involvement of E3 ubiquitin ligases in the regulation of cell cycle [52]. Moreover, it has been shown that TrkA receptors are regulated by the activity of an E3 ubiquitin ligase, Nedd4-2, to modulate neuronal survival [53]. Thus the precise role of RabGEF1 as another E3 ubiquitin ligase in cell cycle control and neuronal survival remains to be determined.

**Fig 9:**
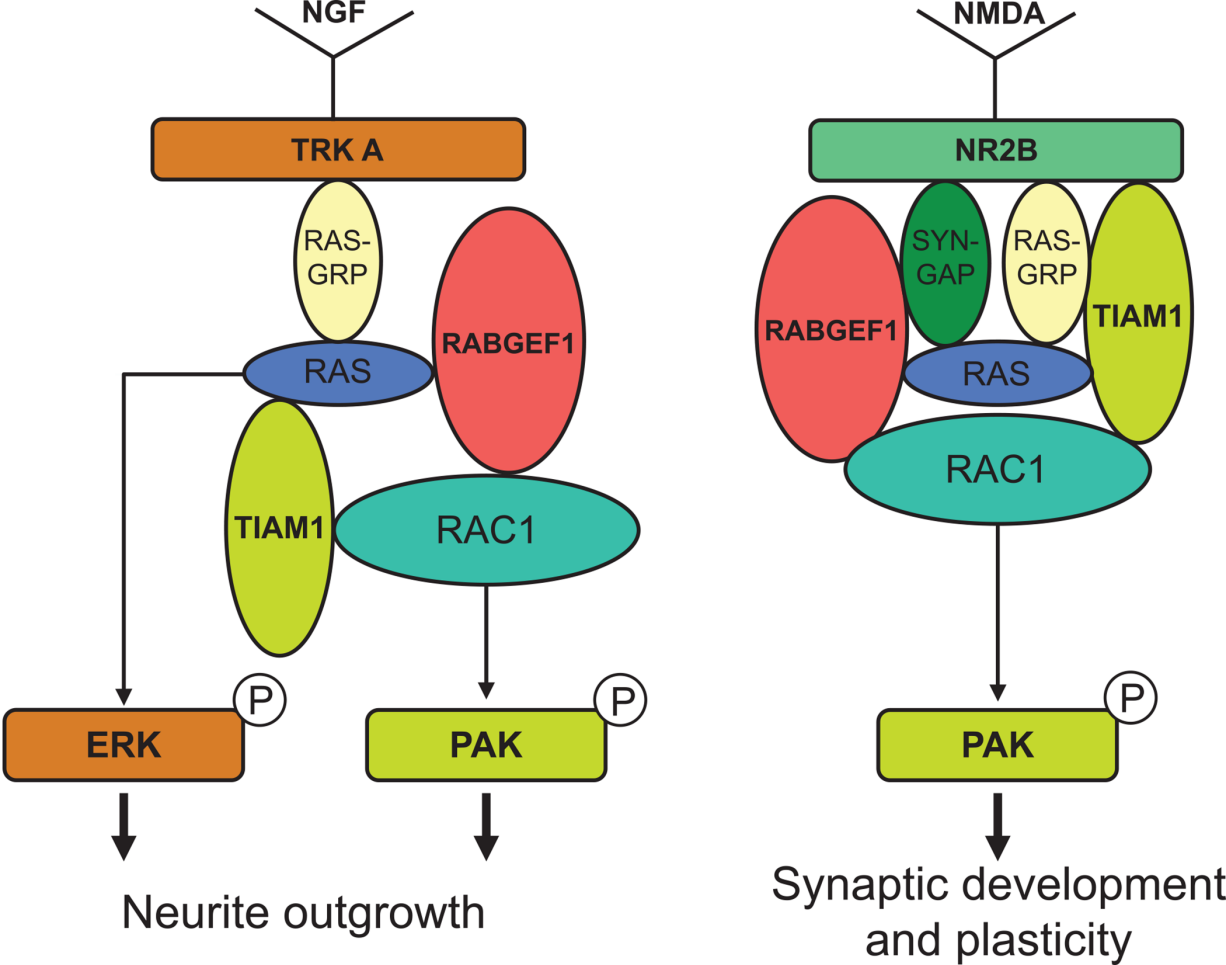
Models illustrating the interaction of RabGEF1 with NGF-induced TrkA-mediated signaling pathways (differentiation) and with NMDA-dependent signaling pathways (neuronal plasticity).

The Rho family of GTPase, such as Rho, Rac1 and Cdc42, are critical signaling components that mediate the effects of NGF in neuronal differentiation [17]. Activation of Rac1 is known to promote actin polymerization in axonal growth and activation of Rho A has been found to inhibit dendritic branch extension and spine formation [17]. In PC12 cells, it has been shown that the neurite outgrowth induced by NGF is mediated by Rac1 activation and RhoA inactivation [23]. In this study, we show that RabGEF1 can εbind to the activated form of Rac1 and negatively regulate the activation of Rac1 and its downstream effector signaling pathways (Fig 5B). These findings are thus consistent with the other data we presented in this study showing that RabGEF1 can negatively regulate NGF-induced neurite outgrowth (Fig 3B–3D). We have previously shown that RabGEF1 negatively regulates Ras activation in FcεRI-dependent activation in mouse mast cells [25]. On the contrary, we show in this study RabGEF1 can also act as a positive regulator for Ras activation in NGF-differentiated PC12 cells (Fig 5A). Taken together, we hypothesize that the exact mechanisms by which RabGEF1 interacts with Ras, or other signaling molecules in particular, are dependent on the cellular context in which the functional activation is induced. Recent studies have shown that RabGEF1 can promote Ras ubiquitination, leading to a reduced ERK activation [32–34]. In the present study, we showed that RabGEF1 can bind to the activated form of Rac1 and negatively regulate the activation of Rac1 and ERK in response to the interaction of a single ligand with its cognate receptor (Figs 5B and 7). Our data thus suggest that Rac1 may serve as another substrate for RabGEF1’s ubiquitin ligase activity besides Ras and that the choice for either substrate may depend on the cellular context in which RabGEF1 exerts its regulatory effects [34]. Indeed, a recent study has shown that the tumor suppressor HACE1, an E3 ubiquitin-ligase, binds preferentially GTP-bound Rac1 and increases the ubiquitylation of active Rac1, leading to the decrease in cellular levels of GTP-bound Rac1 [48]. Therefore, it is conceivable that RabGEF1 can influence Rac1 activity in a similar manner. In this context, it is interesting to note that Tiam1, a Rac1-GEF, can positively regulate Rac1 activation and neurite outgrowth in NGF-differentiated PC12 cells [21]. In addition, activated Ras binds to Tiam1 and such interaction appears to be required for the physical association of Tiam1 and Rac1, which consequently leads to Rac1 activation upon NGF stimulation [21]. It is tempting to speculate that RabGEF1may be part of a signaling interactome which includes Ras, Rac1 and Tiam1 as its components and that they interact in specific manners according to the cellular context of the ligand-receptor interaction that initiates their interaction (Fig 9).

A second line of evidence that is consistent with such network of interaction is provided by a previous study showing that Tiam 1 can interact with NMDA receptors [54]. NMDA receptor stimulation has been shown to induce Tiam1 activation, leading to the Tiam1-dependent activation of Rac1 [54]. In the present study, we showed that RabGEF1 can bind to the NR2B subunit of NMDA receptors and regulate NMDA-mediated functional activation in NGF-differentiated PC12 cells (Fig 8A–8C). Taken together, these findings suggest that RabGEF1 interacts with Tiam1, Ras and Rac1 to form a signaling complex that can respond both to the activation of TrkA receptors to transduce growth and differentiation signals to downstream effector pathways and to the activation of NMDA receptors to mediate functional cellular activation (Fig 9). In this context, the activated form of Ras binds to both Tiam1 and RabGEF1, but Tiam1 functions as a positive regulator of Rac1 activation, whereas RabGEF1 is involved in the negative regulation of Rac1 activation. Therefore, it can be hypothesized that the functional interaction of Tiam1 and RabGEF1 represents parts of a cellular machinery that regulates Rac1 activation through a fine tuning mechanism that is modulated by the interaction of positive and negative control switches.

The relevance of our findings obtained with the PC12 cell model system in this study for the *in-vivo* roles of RabGEF1 in neuronal development and function can be addressed by employing different *in-vivo* experimental approaches. A recent study has examined the roles of RabGEF1 in neuronal development using primary hippocampal neuron cultures with RabGEF1-shRNA transfection. Their data showed that RabGEF1 positively regulates neurite morphogenesis of mouse hippocampal cultured neurons by activating Rab5 and Rab17 [36]. However, such primary cultured neuron approach can suffer many inherent limitations such as inconsistent and unverifiable shRNA-induced knockdown of target protein levels and the possible influence of shRNA expression on cell survival in basal condition. Therefore, further investigations to confirm and fully establish the roles of RabGEF1 in *in-vivo* neuronal development and function can be better achieved using *Rabgef1* conditional knockout mice. The constitutive *Rabgef1* knockout mice have been generated and reported in our original publication on the identification of mouse RabGEF1 [25]. Moreover, our laboratory has recently generated the *Rabgef1* ^*fl/fl*^ (*Rabgef1* ^*2lox*^) mice in which mouse *Rabgef1* exon 2 was flanked by two loxP sites. These mice can serve as the basis for generating specific conditional knockout mouse lines that have RabGEF1 ablated selectively in either postnatal forebrain or postnatal hippocampus, according to the transgenic crossing strategies that were employed in a recent study on the generation of NR2B conditional knockout mice having NR2B ablated in either forebrain or hippocampus [55]. Different conditional *Rabgef1* knockout mouse lines resulted from such breeding strategies will allow direct assessments of the roles of RabGEF1 in *in-vivo* brain development and functions using histological, electrophysiological and behavioral analyses [55].

In summary, our findings suggest that RabGEF1 is a negative regulator of NGF-mediated neurite outgrowth and of NMDA receptor-dependent signaling activation in NGF-differentiated PC12 cells. RabGEF1 appears to exert its effects by influencing the signaling pathways that are known to mediate such cellular processes related to growth/differentiation and receptor-mediated functional responses. We showed that RabGEF1 can bind to Rac1 and negatively regulate its activation induced by NGF stimulation in PC12 cells, suggesting that RabGEF1 may represent a novel and important regulator of Rac1 in the Rac1-dependent signaling pathways that mediate many developmental processes in neurons. On the other hand, we also showed that RabGEF1 can bind to the NMDA receptor NR2B subunit and negatively regulate the signaling responses induced by NMDA stimulation. Our previous studies with mouse mast cells have demonstrated that RabGEF1 is a negative regulator of the signaling and functional responses induced by the interaction of two major distinct receptor systems with their respective cognate ligands in mast cells: activation of the major function-related receptor FcεRI and stimulation of the major growth-related receptor c-Kit [25,27]. The present study further extends these observations by suggesting that RabGEF1 can also regulate specific receptor-mediated function, growth and development of other cell types besides mast cells.

## Supporting Information

S1 Table Partial list of cell cycle proteins whose expressions were influenced by antisense expression of RabGEF1 in PC12 cells stimulated with NGF. Two separate runs of BD PowerBlot Cell Cycle Mini-Screen (BD Biosciences) with 200 cell cycle protein antibodies were used to compare the expression of 200 cell cycle proteins in RabGEF1-AS transfectants stimulated by NGF (100 ng/ml) for 24 hours vs. those in NGF-stimulated RabGEF1-CMV transfectants. Proteins identified by the differential protein array screen are listed in the order of confidence levels (from highest to lowest) as established by the BD PowerBlot Data Analysis software (BD Biosciences).
(DOCX)

S2 Table Partial list of potential and functionally relevant RabGEF1 binding partners in PC12 cells identified by immunoprecipitation/mass spectrometry (MS). Triton-X lysates from PC12 cells that were unstimulated or stimulated with NGF (50 ng/ml) for 30 min or 60 min were subjected to immunoprecipitation with polyclonal anti-RabGEF1 antibody (QCB) [25]. The immunoprecipitated complex coupled to Protein A/G agarose beads was washed in PBS, denatured in 8M urea, and diluted to a final concentration of 1M urea. Trypsin at 1 mg/ml was added at a 1:20 to 1:100 ratio (trypsin:protein) and incubated overnight at 37°C. The beads were then washed in 100% methanol and the solution was lyophilized. The pellet was resuspended in 0.1% TFA prior to LC-MS. The separation of peptides was achieved by reverse phase chromatography using a 30 min gradient on a Dionex LC Packing System followed by analysis on a HCT mass spectrometer (Bruker Daltonics). MASCOT was used to identify proteins from each sample. Proteins identified from the unstimulated or stimulated lysates were subtracted from each other to produce the list of potential binding partners, which are listed below in alphabetical order.
(DOCX)
