# Supplementary material for: RabGEF1/Rabex-5 Regulates TrkA-Mediated Neurite Outgrowth and NMDA-Induced Signaling Activation in NGF-Differentiated PC12 Cells
Source: PLoS One. 2015 Nov 20;10(11):e0142935. doi: 10.1371/journal.pone.0142935 (PMC4654474; doi:10.1371/journal.pone.0142935)
Supplement: S1 Table — Two separate runs of BD PowerBlot Cell Cycle Mini-Screen (BD Biosciences) with 200 cell cycle protein antibodies were used to compare the expression of 200 cell cycle proteins in RabGEF1-AS transfectants stimulated by NGF (100 ng/ml) for 24 hours vs. those in NGF-stimulated RabGEF1-CMV transfectants. Proteins identified by the differential protein array screen are listed in the order of confidence levels (from highest to lowest) as established by the BD PowerBlot Data Analysis software (BD Biosciences). (DOCX) [file pone.0142935.s001.docx]

**Supporting Information**

**S1 Table. Partial list of cell cycle proteins whose expressions were influenced by antisense expression of RabGEF1 in PC12 cells stimulated with NGF.** Two separate runs of BD PowerBlot Cell Cycle Mini-Screen (BD Biosciences) with 200 cell cycle protein antibodies were used to compare the expression of 200 cell cycle proteins in RabGEF1-AS transfectants stimulated by NGF (100 ng/ml) for 24 hours vs. those in NGF-stimulated RabGEF1-CMV transfectants. Proteins identified by the differential protein array screen are listed in the order of confidence levels (from highest to lowest) as established by the BD PowerBlot Data Analysis software (BD Biosciences).

Protein ID Molecular Weight Sign of Change

Cytokeratin 5/8 54/52 +

FEN-1 50 -

JAB1/CSN5 38 -

p27/Kip1 27 +

LAP2 53 -

PCNA 36 -

PTP1C/SHP1 68 -

Rac1 20 -

RBBP 48 -

14-3-3e 29 +

Caveolin 1 22 -

Stat3 92 -

Brm 180 +

Cdk1/Cdc2 34 -

IAK1/Aurora-A Kinase 46 -

eIF-5a 18 +

LAP2 53 -

p36/MAT1 36 -

p38a/SAPK2a 42 -

PTEN 47 -

p190 190 +

Ufd2/E4 146 +

VHL 24 -

Hip1R 120 +

p45/SUG1 45 -
