# Supplementary material for: RabGEF1/Rabex-5 Regulates TrkA-Mediated Neurite Outgrowth and NMDA-Induced Signaling Activation in NGF-Differentiated PC12 Cells
Source: PLoS One. 2015 Nov 20;10(11):e0142935. doi: 10.1371/journal.pone.0142935 (PMC4654474; doi:10.1371/journal.pone.0142935)
Supplement: S2 Table — Triton-X lysates from PC12 cells that were unstimulated or stimulated with NGF (50 ng/ml) for 30 min or 60 min were subjected to immunoprecipitation with polyclonal anti-RabGEF1 antibody (QCB) [25]. The immunoprecipitated complex coupled to Protein A/G agarose beads was washed in PBS, denatured in 8M urea, and diluted to a final concentration of 1M urea. Trypsin at 1 mg/ml was added at a 1:20 to 1:100 ratio (trypsin:protein) and incubated overnight at 37°C. The beads were then washed in 100% methanol and the solution was lyophilized. The pellet was resuspended in 0.1% TFA prior to LC-MS. The separation of peptides was achieved by reverse phase chromatography using a 30 min gradient on a Dionex LC Packing System followed by analysis on a HCT mass spectrometer (Bruker Daltonics). MASCOT was used to identify proteins from each sample. Proteins identified from the unstimulated or stimulated lysates were subtracted from each other to produce the list of potential binding partners, which are listed below in alphabetical order. (DOCX) [file pone.0142935.s002.docx]

**Supporting Information**

**S2 Table. Partial list of potential and functionally relevant RabGEF1 binding partners in PC12 cells identified by immunoprecipitation/mass spectrometry (MS).** Triton-X lysates from PC12 cells that were unstimulated or stimulated with NGF (50 ng/ml) for 30 min or 60 min were subjected to immunoprecipitation with polyclonal anti-RabGEF1 antibody (QCB) [25]. The immunoprecipitated complex coupled to Protein A/G agarose beads was washed in PBS, denatured in 8M urea, and diluted to a final concentration of 1M urea. Trypsin at 1 mg/ml was added at a 1:20 to 1:100 ratio (trypsin:protein) and incubated overnight at 37^o^C. The beads were then washed in 100% methanol and the solution was lyophilized. The pellet was resuspended in 0.1% TFA prior to LC-MS. The separation of peptides was achieved by reverse phase chromatography using a 30 min gradient on a Dionex LC Packing System followed by analysis on a HCT mass spectrometer (Bruker Daltonics). MASCOT was used to identify proteins from each sample. Proteins identified from the unstimulated or stimulated lysates were subtracted from each other to produce the list of potential binding partners, which are listed below in alphabetical order.

BING4 protein

Cyclin D1

GAP-associated protein p190

Keratin 19

Mitogen activated protein kinase kinase kinase 1

N-methyl-D-aspartate receptor-N2B subunit

Neurofibromatosis 1

Neuron-glia-CAM-related cell adhesion molecule

Phospholipase C-γ2

Proline rich synapse associated protein 1

Protein-tyrosine-phosphatase, non-receptor type 11

Rho-associated kinase β

Semaphorin 6C

SH2-containing inositol phosphatase 2

Synapse-associated protein 90/postsynaptic density-95-associated protein 3

Synaptic density protein PSD-3

Synaptopodin

Synaptotagmin 11

SynGAP

TBP-interacting protein Tip 120B

Transient receptor potential channel 4-β

Zn finger protein 291
